# The Impact of Standard Cross-Linking on the Corneal Optical Density–Age Relationship in Keratoconus After Mechanical Stripping of the Epithelium

**DOI:** 10.1155/2024/8827837

**Published:** 2024-10-15

**Authors:** Maja Bohac, Fanka Gilevska, Alma Biscevic, Ivan Gabric, Kresimir Gabric, Sudi Patel

**Affiliations:** ^1^Specialty Eye Hospital Svjetlost, School of Medicine, University of Rijeka, Zagreb, Croatia; ^2^Eye Clinic Sistina Oftalmologija, Skopje, North Macedonia

**Keywords:** age, corneal optical density, corneal tomography, cross-linking, keratoconus

## Abstract

**Background:** To determine if cross-linking (CXL) treatment modifies any pre-existing association between corneal optical density (COD) and age in keratoconus free of corneal scarring.

**Methods:** COD was monitored in two groups (i) before and after standard CXL treatment for keratoconus (de-epithelization with a crescent blade, *n* = 69 eyes) and (ii) age/gender-matched cases without any signs of keratoconus (*n* = 24 eyes). Seven different markers of COD were quantified using a 0–100 grey scale, supplied with Pentacam™ software.

**Results:** Mean age (±sd, range) in Group I (19 females and 50 males) was 24.2 years (±7.2, 11–44) and in Group II (9 females and 15 males), it was 24.7 years (±7.6, 17–45). COD over the apex and along the depth of the cornea (y, arbitrary scale units) was associated with age (*x*, in years) in Group I at preop (*y* = 0.08*x* + 13.12, *r*_*s*_ = 0.350 and *p*=0.003), at 12 months postop (*y* = 0.08*x* + 15.15, *r*_*s*_ = 0.295 and *p*=0.014) and in Group II, at the start (*y* = 0.16*x* + 11.28, *r*_*s*_ = 0.474 and *p*=0.019) and 12 months later (*y* = 0.24*x* + 8.63, *r*_*s*_ = 0.600 and *p*=0.002). The change in COD following CXL was significantly associated with the preop value.

**Conclusion:** CXL initially breaks down the pre-existing relationship between COD and age. This is re-established by 12 months postop. The CXL induced change in COD depends on the preop value but not on the patient's age.

## 1. Introduction

The aged cornea tends to be thinner [1–8], exhibits lower cell densities [9–17], has greater light scattering properties [18] and different structural characteristics when compared with the younger counterpart [19–22]. Furthermore, confocal microscopy has revealed that the concentration of microdot-like structures visible in the stroma is reduced in the older cornea [23, 24]. It is reasonable to assume that a change in any of these features of the cornea carries the potential to affect corneal transparency. The backscattering properties of the cornea can be readily observed using techniques designed to estimate the optical density of the cornea, and a few clinical studies have reported higher corneal optical density (COD) values in older subjects [25–32]. A link between COD and age has been reported in keratoconus with Down's syndrome [27]. COD values are mostly elevated in keratoconus cases free of any corneal scarring when compared with age-matched controls [28, 33–36]. After a literature search, it remains unknown if COD values are age-related in keratoconus free of Down's syndrome. COD increases after cross-linking (CXL) treatment of keratoconus and gradually regresses towards pretreatment levels [37–49]. Recent reports on the coefficient of variation in COD values range from 2% to 4% in healthy corneas, 3% to 5% in keratoconus and 7% to 15% in CXL treated cases [50]. The repeatability of COD estimation tends to be poorer in keratoconus compared to the normal control group particularly within the central 2-mm zone [26, 33, 50, 51]. Furthermore, the measurement of COD may be affected by ambient light condition. Thus, it would be useful to verify the repeatability of COD measurements obtained from a single device, under different ambient light conditions, when investigating the effect of age on COD. The aim of this study was to assess the repeatability of COD measurements and determine if CXL treatment impacts on any association between COD and age in keratoconus.

## 2. Subjects and Methods

### 2.1. Measurement of COD

COD was assessed using Pentacam HR (Oculus, Optikgeräte GmbH, Wetzlar, Germany). The instrument's Scheimpflug image processing software includes an option to create a display of optical density values within and over the cornea [52]. The software averages and displays the optical density at the apex (within the central 2-mm zone) and three concentric zones (within paracentral 2–6 mm and 6–10 mm regions and peripheral 10–12 mm region), in three separate layers along the depth of the cornea (anterior 120 μ, posterior 60 μ and remaining central bed). Optical density values are relative and displayed as percentage figures ranging from 0 (nil backscatter of incident rays) to 100 (total backscatter of all incident rays) along an arbitrary grey scale for each of the 12 regions. The software also averages the total optical density along the depth of the cornea within the apex, each concentric zone and the whole cornea. CXL does not appear to exert a significant effect on COD values in the peripheral cornea (6–10 mm and 10–12 mm annuli) and posterior (60 μ) sector [37, 40, 41, 43, 45]. So COD values for the corneal apex (covering the anterior [0–2ant] and central layers [0–2cent)], paracentral (2–6 mm region covering the anterior [2–6ant] and central layers [2–6cent]), total anterior (Tot Ant) region layer, total central layer (Tot Cent) and whole cornea (Tot) were harvested during this study. Details of the various descriptors of COD are summarised in Table 1.

### 2.2. Pilot Study for Repeatability of COD Measurements

Under dark conditions (≈8 to 10 lux, checked using a standard light metre, UT383S, UNI-T, Tipa, spol. r.o. Sadová, Czech Republic), ten consecutive COD measurements were taken from one healthy cornea and one case of keratoconus. This was repeated 5 min later under light conditions (≈200 lux with room lights switched on). The intersessional variability was estimated by repeating the whole process 1 week later for the healthy cornea.

The results would indicate which light conditions (dark conditions ≈8 to 10 lux or light condition ≈200 lux) were more suitable for the main clinical study.

### 2.3. Design of Clinical Investigation

This was a prospective, consecutive, observational study on patients treated for keratoconus by CXL. The study was approved by the Ethical Board Committee of Institution where the research was conducted, and the tenets of the Declaration of Helsinki were respected on all occasions. Signed consent was obtained from each patient after the aims, and procedures associated with the study were fully explained.

### 2.4. Initial Assessment and Indications for CXL

All keratoconus patients scheduled for CXL were previously diagnosed using the “ABCD” Belin grading system [53, 54]. In these patients, the astigmatism had increased by at least 1D according to subjective refraction and/or corneal surface topography over the previous year. Cases with any scarring visible during slit-lamp examination, history of ocular surgery, infections, other corneal diseases, corneas thinner than 380μ or any systemic conditions that may affect corneal clarity were excluded.

Each patient underwent an ophthalmological examination on the first and all follow-up visits. The examination included assessment of CDVA, refraction, tonometry, corneal characteristics using Pentacam HR, biomicroscopy, dilated fundus examination, corneal topography, thickness distribution and COD values were captured for later analysis.

### 2.5. CXL Procedure and Postoperative Management

The CXL procedure adhered to the standard Dresden protocol. Preoperatively, all the patients received topical anaesthetic (Tetracaine HCL 0.5%, Alcon Forth Worth, Texas) and miotic (Isopto Carpine 2% HCl, Alcon, Forth Worth, Texas) drops. The pupil was constricted to decrease the amount of UVA radiance to the crystalline lens and retina in order to prevent any possible damage of it to those structures. The fornices and the periocular region were cleaned with povidone 10% (Betadine 10%, Alcaloid, Skopje). The central 8.5–9.0-mm diameter of the corneal epithelium was removed with a crescent knife. Intraoperative pachymetry was measured with an ultrasound handheld pachymetre (Pocket II, Quantel Medical, Cournon d'Auvergne, France). Riboflavin (Peschke D, Peschke Trade, Huenenberg, Switzerland) was applied every 3 min for the next 30 min. The cornea was subjected to UV-A radiation with a wavelength of 370 nm and energy of 3 mW using UVA CXL lamp (VEGA CBM-X-Linker, Carleton Optical, Chesham, United Kingdom) for 30 min (6 cycles of 5 min each). The correct irradiance density (between 2.7 mW/cm^2^ and 3.3 mW/cm^2^) and dose (5.4 J/cm^2^) were checked before commencing every CXL procedure [14]. After the irradiation, topical atropine 1% (Atropine Sulphate, Cooper, Athens, Greece) and antibiotic (Tobrex, Alcon, Forth Worth, Texas) drops were applied, and a bandage soft contact lens (Air Optix Night and Day Aqua, Alcon, Forth Worth, Texas) was placed over the cornea.

Postoperative therapy included a combination of antibiotic/corticosteroid drops (Tobradex, Alcon, Forth Worth, Texas) instilled four times daily for the next two weeks afterwards the drops were tapered off gradually over six weeks.

Postoperative examinations were scheduled at 1, 4 and 7 days, then at 1, 3, 6 and 12 months. Thereafter, the patients were advised to have a full ophthalmological examination on an annual basis.

### 2.6. Normal Control Group

An age- and gender-matched group of individuals was recruited as a normal control group. These individuals were confirmed as keratoconus-free according to “ABCD” Belin grading system [53, 54], had clear corneas, no signs of any scarring on slit-lamp examination and was without any signs of ectasia topographic or tomographic irregularities or health conditions known to affect corneal characteristics.

### 2.7. Data Collection and Analysis

COD values obtained at preop, 1, 2, 3, 6 and 12 months for cases that underwent unremarkable CXL were used for an analysis. Only the values obtained from the right eyes were included from bilateral cases.

In the control group, the COD values were obtained at the start of the study and 12 months later. Data were analysed to determine if there was a correlation between the following:

i. Subject age and density values at each stage of the investigation
ii. The change in the density value and preop density values in the treated cases (Spearman's rank correlation coefficient, *r*_*s*_)

If density values are age-related in the cohort of subjects, and then the analysis would be extended to determine if (a) apparent differences between age–density relationships were significant (Wald test for significance of differences between two slopes [55]) and (b) changes in density values resulting from CXL were age-related. The significance level was set at *p* < 0.05.

## 3. Results

### 3.1. Pilot Study for Repeatability of COD Measurements

In both the keratoconus and control, the values obtained under dark conditions were mostly lower compared with values obtained under light conditions (*p* < 0.01) for each descriptor noted in Table 1. Under dark conditions, some of the COD values were higher in keratoconus. Under light conditions, the COD values were consistently higher in keratoconus compared with the control (*p* < 0.01). Consequently, it was decided to discontinue acquiring data under dark conditions. There were insignificant intersessional differences in five of the seven descriptors of COD (Table 1).

### 3.2. Clinical Investigation

The CXL group consisted of 19 female and 50 male (69 eyes) subjects of mean age (±sd, range) 24.2 years (±7.2, 11–44). The normal control group consisted of 9 females and 15 males of mean age (±sd, range) 24.7 years (±7.6, 17–45). All COD values were obtained under light conditions (≈200 lux).

#### 3.2.1. COD and Age

In both groups, there were some significant correlations between age and COD values at the start and 12 months later. The best fit expressions describing these associations are shown in Table 2. In each group, the apparent change in the slope over the year was not significant (*p* > 0.05). The difference in slope values between the groups was not significant (*p* > 0.05 for all possible comparisons).

In the treated group, there were no significant correlations between COD and age at 1, 3 and 6 months postop.

#### 3.2.2. Change in Corneal Density in the Treated Group

In the treated cases (*n* = 69), the change in density (Δ = preop value–postop value at 12 months) was associated with the corresponding preop value for 2–6 Cent (*r*_*s*_ = 0.331 and *p*=0.006), Tot Ant (*r*_*s*_ = 0.379 and *p*=0.005), Tot Cent (*r*_*s*_ = 0.339 and *p*=0.002) and tot (*r*_*s*_ = 0.397 and *p*=0.006) markers of density.

The ratio, Δ/preop value, was associated with the preop density value for 2–6 Ant (*r*_*s*_ = 0.316, *p*=0.008), 2–6 Cent (*r*_*s*_ = 0.382, *p*=0.001), Tot Ant (*r*_*s*_ = 0.443, *p* < 0.001), Tot Cent (*r*_*s*_ = 0.439, *p* < 0.001) and Tot (*r*_*s*_ = 0.421, *p* < 0.001). The best fit expressions describing the significant associations between Δ/preop and preop values are shown in Table 3. There were no significant correlations between age and Δ.

#### 3.2.3. Change in COD in the Normal Control Group

In the normal control group (*n* = 24), the difference in density (Δ = value at the start of study–value at 12 months) was associated with the corresponding value at the start of the study for 0–2 Ant (*r*_*s*_ = 0.536 and *p*=0.007), 2–6 Ant (*r*_*s*_ = 0.441 and *p*=0.031) and Tot Ant (*r*_*s*_ = 0.668 and *p* < 0.001). The ratio Δ/value at the start of study was associated with the density value at the start of the study for 0–2 Ant (*r*_*s*_ = 0.490, *p*=0.015) but not for the remaining descriptors (*p* > 0.05). There were no significant correlations between age and Δ.

## 4. Discussion

The COD values generated by Pentacam are not absolute values. These are relative values and dependent upon the factory calibration procedures. An artificial cornea could be constructed to test the validity of the instrument. However, a more authentic evaluation of the reliability of the instrument in a real-world clinical setting would be gained by checking a natural living cornea. Table 1 shows there were significant intersessional differences in just two of the seven markers of COD. The intersessional variability was greatest for the anterior apical region of the cornea (0–2 Ant), and this validates earlier reports [50]. The average s.d. value was ±0.53 units, equivalent to ±4% of the optical density, along the entire depth of the cornea over the central 6 mm apex (Tot).

**Figure 1:**
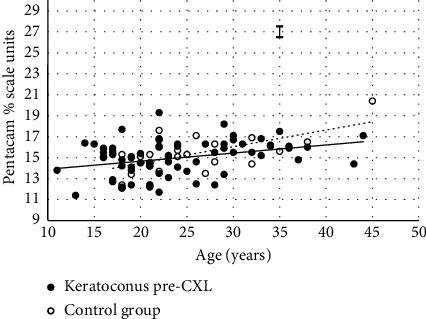
COD within the central 6-mm apex and along the entire depth of the cornea in relation to the subject age at the start of the study. Filled circles are keratoconus subjects before CXL. The association between the subject age (*x*) and the arbitrary COD value (*y*) is best represented by the solid line where *y* = 0.08*x* + 13.12 (*r*_*s*_ = 0.350, *n* = 69 and *p*=0.003). Empty circles are normal, control and subjects. The association between the subject age (*x*) and the arbitrary COD value (*y*) is best represented by the hatched line where *y* = 0.16*x* + 11.28 (*r*_*s*_ = 0.474, *n* = 24 and *p*=0.019). Ɪ = ±s Keratoconus d error bar represents the typical test-retest repeatability in measurement of COD.

The optical density of the cornea was age-related, and this adds further support to previous reports [25–32]. The expressions in Table 2 and Figure 1 show that the relationships between COD and age were similar in both groups at the start of the study.

Significant correlations were not detected in four discrete regions of the cornea (0–2ant, 0–2cent, 2–6ant and 2–6cent). Clearly, the association between age and optical density in the central 6-mm zone was not confined to specific sites within the cornea. The mechanism underpinning the gradual loss of corneal clarity within the older keratoconic cornea is on par with the mechanism operating in the normal healthy cornea. The chain of events leading to keratoconus does not have a noticeable impact on the ageing process affecting corneal clarity. CXL initially compromised the association, but it was recovered by 12 months. However, the association recovered for the descriptors of optical density covering the entire depth of the apical 6-mm zone of the cornea (Tot) and central region (Tot cent) but not for the anterior region (Tot Ant). Salman et al. [56] followed up keratoconus patients aged 8–18 years that had undergone CXL treatment. The age of the youngest patient in our study was 11 years. It is questionable whether a significant correlation between age and the Tot Ant factor might have been detected at 12 months postop if younger cases had been enrolled. However, according to one power equation, the minimum number of cases that should be enrolled to determine if a significant correlation between age and the Tot Ant descriptor is re-established by 12 months after CXL is 215 [57].

**Figure 2:**
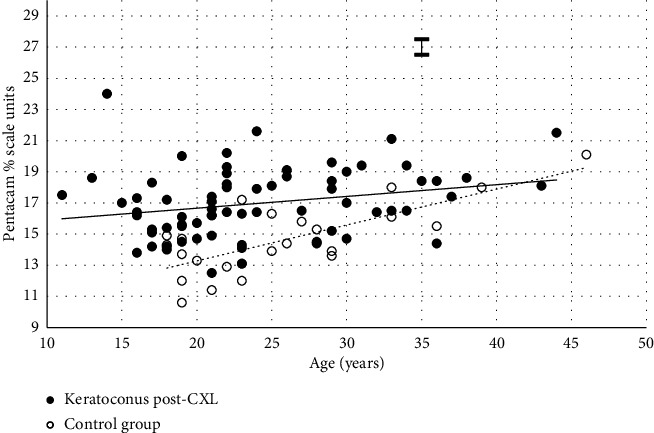
COD within the central 6 mm apex and along the entire depth of the cornea in relation to subject age at 1 year. Filled circles = keratoconus subjects after CXL. The association between subject age (*x*) and the arbitrary COD value (*y*) is best represented by the solid line where *y* = 0.08*x* + 15.15 (*r*_*s*_ = 0.295, *n* = 69 and *p*=0.014). Empty circles = normal, control, subjects. The association between subject age (*x*) and the arbitrary COD value (*y*) is best represented by the hatched line where *y* = 0.23*x* + 8.67 (*r*_*s*_ = 0.600, *n* = 24 and *p*=0.002). Ɪ = ±sd error bar represents the typical test–retest repeatability in measurement of the COD.

Figure 1 shows the two groups had similar optical density–age relationships at the start of the study.  Figure 2 shows the similarity between two groups was broken down by 12 months. Turning to the expressions in Table 2, there are apparent differences between the two groups at 12 months, but these were not statistically significant.

Consejo et al. [30] claimed that inadvertent tilting of the eye during COD assessment with Pentacam can affect the results obtained. Nevertheless, they still reported an association between COD values and age after correcting for tilt. Changes in tilt occurring after CXL may have influenced the COD values and contributed to the differences in the slope values in Figure 2. In both groups, the change in COD over the period (Δ = value at the start of the study minus value at 12 months) was not age-related. However, the value of Δ correlated with the respective preop value of the 2–6 Cent, Tot Ant, Tot Cent and Tot markers in the keratoconus group.

Similar trends were also encountered in the control group where Δ was associated with the value at the start of the study for the 0–2 Ant, 2–6 Ant and Tot Ant markers. The suggestion is that these specific markers are of questionable value when used to monitor COD in healthy corneas.

**Figure 3:**
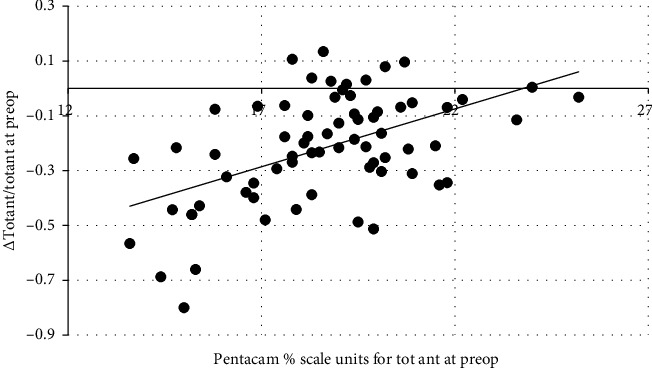
Optical density of the cornea within the anterior 120 μ depth, and over the central 6 mm apex, of the cornea at preop (*x*) in comparison to proportion of change observed at 1 year after CXL. Δ = *x* minus optical density at 1 year and *y* = the ratio Δ/*x*. The association between *x*, the arbitrary COD at preop and the ratio *y* is best represented by the solid line where *y* = 0.04*x* − 1.01 (*r*_*s*_ = 0.443, *n* = 69 and *p*=0.00014).

Table 3 shows five of the seven markers of COD were correlated with the changes observed at 12 months postop.  Figure 3 shows the data depicting changes in the Tot Ant marker are mainly below the “0, 0” abscissa. These are cases where the optical density increased resulting in the cornea becoming less clear compared with preop levels. The small number of cases above the abscissa are those where clarity improved in comparison with preop levels. Furthermore, Figure 3 shows that when the preop Tot Ant value is below 24, then the corneal clarity is expected to become worse at 12 months postop. According to the expressions in Table 3, the relatively clearer cornea associated with less severe keratoconus is more liable to experience a greater percentage loss in clarity by 12 months after CXL. Equation 5 in Table 3 predicts that the Tot marker will increase when the preop value is ≤ 19. Turning to Figure 1, only one of the 69 keratoconus cases had a Tot marker value of >19.

CXL eliminates the structural differences in the corneal anatomy that result in age-related variations in optical density. The re-establishment of this relationship by 12 months postop is indicative of a gradual realignment of the corneal ultrastructure towards preop levels. This cannot be said for the anterior 120 μ section of the cornea. Cohen et al. [55] considered the various mechanisms leading to loss and recovery of corneal clarity following surgical intervention. Regions where corneal haze persist are, besides other factors, associated with disorganised extracellular matrix and higher concentrations of myofibroblasts. The bulk of data points below the abscissa in Figure 3 direct us to accept that, in most cases, the cornea had not recovered to preop levels by 12 months after CXL [58].

### 4.1. Limitations of the Study

The results are limited by the characteristics of the cases enrolled for treatment. Only cases of progressive keratoconus were approved for CXL. The outcomes might have been different if CXL treatment had been provided earlier before progression was confirmed [59, 60].

The study is further limited by the number of cases, period of the follow-up, the CXL procedure and the procedure used to assess COD. However, the data predicts at least 215 cases should be followed up for 12 months to determine if the association between age and Tot Ant is re-established by 12 months postop.

## 5. Conclusion

In keratoconus, the optical density of the cornea is age-related, and this association breaks down after corneal CXL with mechanical stripping of the epithelium. The association is partially re-established by 12 months postop. Age is not a factor associated with the change in COD following CXL. However, the changes in some descriptors of COD after CXL are dependent upon the values recorded at preop. The 0–2 Ant, 2–6 Ant and Tot Ant markers of COD are of limited value when used to monitor COD in control groups.

## Figures and Tables

**Table 1:** Intersessional variability of COD measurements obtained under light conditions in a single healthy control.

	**Session 1**	**Session 2**	

0–2ant	21.4 (±1.33, 20.5–22.4)	24.1 (±1.81, 22.8–25.5)	*p*=0.006
0–2cent	14.3 (±0.29, 14.0–14.5)	14.2 (±0.35, 13.9–14.4)	*p*=0.621
2–6ant	12.9 (±0.63, 12.5–13.4)	12.8 (±0.19, 12.7–12.9)	*p*=0.687
2–6cent	12.1 (±0.11, 12.0–12.2)	11.9 (±0.17, 11.8–12.0)	*p*=0.006
Tot ant	16.8 (±0.50, 16.4–17.1)	17.2 (±0.57, 16.7–17.6)	*p*=0.153
Tot cent	14.3 (±0.24, 14.1–14.5)	14.6 (±0.43, 14.2–14.9)	*p*=0.180
Tot	14.5 (±0.28, 14.3–14.7)	14.7 (±0.46, 14.4–15.0)	*p*=0.192

*Note:* COD measurements were recorded from the following regions: 0–2ant = central 2 mm apex and along the anterior 120 μ depth of the cornea. 0–2cent = central 2 mm apex and along the mid-section, excluding anterior 120 μ and posterior 60 μ sections of the cornea. 2–6ant = 2–6 mm paracentral annulus and along the anterior 120 μ depth of the cornea. 2–6cent = 2–6 mm paracentral annulus and along the mid-section, excluding anterior 120 μ and posterior 60 μ sections of the cornea. Tot ant = central 6 mm apex and along the anterior 120 μ depth of the cornea. Tot cent = central 6 mm apex and along the mid-section, excluding anterior 120 μ and posterior 60 μ sections of the cornea. Tot = central 6 mm apex along the entire depth of the cornea. Mean (±sd, 95% CI limits) of the Pentacam-generated density values are in percentage grey scale units ranging from 0 (totally clear) to 100 (totally opaque). The intersessional differences were not significant for 0–2cent, 2–6ant, tot ant, tot cent and tot descriptors (paired *t*-test *p* > 0.05) but were significant for 0–2ant and 2–6cent (*p* < 0.05) descriptors.

**Table 2:** Correlations between corneal density (*y*) and age (*x*) at the start of the study and at 12 months.

**CXL-treated group (*n* = 69)**		
	**Pre-CXL**	**12 months post-CXL**

Tot ant	*y* = 0.10*x* + 16.29, *r*_*s*_ = 0.283 and *p*=0.018	*y* = 0.09*x* + 20.73, *r*_*s*_ = 0.178 and *p*=0.144
Tot cent	*y* = 0.07*x* + 12.74, *r*_*s*_ = 0.347 and *p*=0.004	*y* = 0.08*x* + 14.13, *r*_*s*_ = 0.341 and *p*=0.004
Tot	*y* = 0.08*x* + 13.12, *r*_*s*_ = 0.350 and *p*=0.003	*y* = 0.08*x* + 15.15, *r*_*s*_ = 0.295 and *p*=0.014

**Control group (*n* = 24)**		
	**Start**	**12 months**

Tot ant	*y* = 0.16*x* + 14.86, *r*_*s*_ = 0.412 and *p*=0.046	*y* = 0.28*x* + 10.53, *r*_*s*_ = 0.645 and *p* < 0.001
Tot cent	*y* = 0.16*x* + 10.84, *r*_*s*_ = 0.430 and *p*=0.036	*y* = 0.23*x* + 8.17, *r*_*s*_ = 0.607 and *p*=0.002
Tot	*y* = 0.16*x* + 11.28, *r*_*s*_ = 0.474 and *p*=0.019	*y* = 0.24*x* + 8.63, *r*_*s*_ = 0.600 and *p*=0.002

*Note:* Significant correlations were not detected between age and 0–2ant, 0–2cent, 2–6ant and 2–6cent in either group except for the correlation with 2–6cent in the control group at 12 months (*r*_*s*_ = 0.475 and *p*=0.019). In the treated group, the 95% confidence intervals of the slopes in the expressions range from ±0.05 to ±0.08, and there was a significant correlation between age tot ant at preop but not at 12 months postop. In the control group, the 95% confidence intervals for the slopes at the start and 12 months later were, respectively, tot ant ±0.16 and ±0.12, tot cent ±0.09 & ±0.09 and tot ±0.08 and ±0.09.

**Table 3:** Correlations between COD at preop (*x*) and ratio (*y*) at 12 months of the change in the COD/preop value in the treated group.

2–6ant	*y* = 0.04*x* − 0.99, *r*_*s*_ = 0.316 and *p*=0.008, equation 1
2–6cent	*y* = 0.06*x* − 0.97, *r*_*s*_ = 0.382 and *p*=0.001, equation 2
Tot ant	*y* = 0.04*x* − 1.00, *r*_*s*_ = 0.443 and *p* < 0.001, equation 3
Tot cent	*y* = 0.05*x* − 0.87, *r*_*s*_ = 0.439 and *p* < 0.001, equation 4
Tot	*y* = 0.05*x* − 0.91, *r*_*s*_ = 0.421 and *p* < 0.001, equation 5

*Note: y* = (*x* minus postop value at 12 months)/*x*. Significant correlations were not detected between preop and change in COD/preop value at 12 months for 0–2ant and 0–2cent descriptors. The expressions predict COD is expected to increase (i.e., the cornea becomes more opaque and less clear) when the preop value of either 2–6ant ≤ 23, or 2–6cent ≤ 16, or tot ant ≤ 24, or tot cent ≤ 17, or tot ≤ 19. In the control group, significant correlations were not revealed between COD at the start of the study and change in COD/preop value, at 12 months except for 0–2ant (*r*_*s*_ = 0.490 and *p*=0.015).

## Data Availability

The data used to support the findings of this study are available from the corresponding author upon request.
